# Shuxuening injection, derived from *Ginkgo biloba* leaf, induced pseudo-allergic reactions through hyperactivation of mTOR

**DOI:** 10.1080/13880209.2020.1784238

**Published:** 2020-07-02

**Authors:** Lianmei Wang, Jingzhuo Tian, Suyan Liu, Yanyan Zhang, Jing Liu, Yan Yi, Chunying Li, Yong Zhao, Yushi Zhang, Jiayin Han, Chen Pan, Guiqin Li, Zhong Xian, Aihua Liang

**Affiliations:** a Key Laboratory of Beijing for Identification and Safety Evaluation of Chinese Medicine, Institute of Chinese Materia Medica, China Academy of Chinese Medical Sciences, Beijing, China; b Traditional Chinese Medicine Injection Innovation Center, Shijiazhuang, Hebei Province, China

**Keywords:** Vascular endothelial cell hyperpermeability, VEGF, rapamycin

## Abstract

**Context:**

Shuxuening injection (SXNI), derived from the leaf of G*inkgo biloba* L. (Ginkgoaceae), is widely used to treat cardio-cerebral vascular system related disease due to the efficacy of dilating the blood vessels and improving the function of microcirculation. Nevertheless, SXNI induces immediate hypersensitivity reactions in clinics and the molecular mechanisms are unknown.

**Objective:**

The present study investigates the molecular mechanism of SXNI mediated hypersensitivity reactions.

**Materials and methods:**

Naive male ICR mice (*n* = 10) were administered (i.v.) with negative control combined with Evans blue (EB) (CTL-EB), SXNI (14 or 70 mg/kg) combined with EB (SXNI/1-EB or SXNI/4-EB), vascular leakage was evaluated, ears and lungs were collected for histopathological analysis. *In vitro*, TSC1 was knockdown in human umbilical vein endothelial cells (HUVECs). HUVECs were incubated with SXNI, and the alterations of endothelial cell permeability were observed. Rapamycin (mTOR inbibitor) was used to investigate SXNI-induced hypersensitivity reactions both in mice and HUVECs.

**Results:**

SXNI (70 mg/kg) induced vascular leakage in mice. Slight oedema and microvascular dilation in the ears, and broaden of alveolar septal and monocyte infiltration in the lungs were observed in SXNI (70 mg/kg) treated mice. mTOR inhibitor alleviates SXNI mediated vascular endothelial hyperpermeability both *in vitro* and *in vivo*.

**Discussion and conclusions:**

SXNI stimulates pseudo-allergic reactions through hyperactivation of mTOR signalling pathway. Our work provides the new molecular mechanism of drug related pseudo-allergic reactions, and a potential drug to prevent and treat SXNI mediated hypersensitivity reactions.

## Introduction

Shuxuening injection (SXNI) is derived from the leaf of *Ginkgo biloba* L. (Ginkgoaceae), and the effective components are flavonoids, bilobalide and ginkgolides (Wang et al. 2019). SXNI is widely used to treat ischaemic cardiovascular and cerebrovascular diseases, coronary heart disease, angina pectoris, cerebral embolism and cerebral vasospasm diseases clinically in China (Wang et al. 2019; Cao et al. 2020). Dilation of the blood vessels and function improvement of the microcirculation are the main efficacy of SXNI. However, the adverse drug reactions (ADRs) are reported approximate 5.84% in SXNI treated patients based on meta-analysis (Wang et al. 2018). Retrospective analysis revealed that 68.4% of SXNI mediated ADRs showed allergic clinical manifestation (Mao et al. 2016). We previously reported that SXNI induced non-IgE mediated hypersensitivity reactions through active anaphylaxis test (ASA) and passive cutaneous anaphylaxis (PCA) tests (Yi et al. 2017). However, the underlying molecular mechanisms of SXNI induced hypersensitivity reactions were unknown.

Pseudo-allergic reactions also called anaphylactoid reactions, are activated at the first exposure to simulators and non-IgE mediated allergic reactions with indistinguishable clinical manifestations including urticaria, angioedema, anaphylactoid reactions, rhinitis, asthma anaphylaxis, bronchospasm and gastrointestinal disorder with allergic reactions (He et al. 2013; Pichler 2019). The clinical symptoms associated with SXNI hypersensitivity reactions contain urticaria, erythema, allergic reaction of digestive system, and difficult breathing. All of these symptoms are related to the elevation of vascular leakage (Mao et al. 2016).

Vascular permeability plays pivotal role in keeping the health status of normal tissues, and elevates in the pathologies including acute inflammation, wounds, tumour angiogenesis, and chronic inflammatory diseases (Nagy et al. 2002, 2003, 2012). Vascular permeabilizing factors containing VEGF, histamine, serotonin and platelet activating factor (PAF) induce acute vascular permeability (Boesiger et al. 1998; Nagy et al. 2012; Ono et al. 2017). We previously reported a mouse model to evaluate drug (injections) induced pseudo-allergic reactions due to vascular leakage (Han et al. 2016, 2018; Pan et al. 2019).

Mechanistic target of rapamycin (mTOR) sensing nutrient and energy in cell, modulates cell growth and survival through regulating anabolic processes, protein synthesis and autophagy (Ma et al. 2010; Chen et al. 2014; Ma et al. 2014). mTOR is involved in the innate and adaptive immune response (Sinclair et al. 2017; Zou et al. 2019). Studies have uncovered that mTOR elevate the expression of VEGF (Brugarolas et al. 2003; El-Hashemite et al. 2003; Karar and Maity 2011). However, whether mTOR-VEGF functions in the drug related pseudo-allergic reactions is still not clear.

In this study, we investigated the molecular mechanism of SXNI mediated hypersensitivity reactions. In addition, our work provides the potential candidate target for the prevention and treatment of SXNI hypersensitivity reactions in clinics.

## Materials and methods

### Animals

Male ICR mice (23–25 g) were obtained from Charles River Company, Beijing, China. Mice were maintained in cages in a room equipped with an air-filtering system, and they were kept on a 12 h light/dark cycle. The animals were fed standard food and given sterilized water.

This study was carried out in strict accordance with the recommendations of ethical guidelines and regulations for the use of laboratory animals and cells issued by the Institute of Chinese Materia Medica, China Academy of Chinese Medical Sciences, Beijing, China. All animal-related protocol was approved by the Committee on the Ethics of Animal Experiments of the Institute of Chinese Materia Medica, China Academy of Chinese Medical Sciences.

### Reagents

SXNI was obtained from Shineway pharmaceutical (China). Evans blue (EB), rapamycin, FITC-dextran and rhodamine-phalloidin were obtained from SigmaAldrich (USA). TSC1, pS6 was from Cell signalling technology (USA). VEGF was from Millipore Corporation (USA), Actin was from Santa cruz biotechnology (USA). VEGF Elisa kit was from R&D systems (USA). DMEM, foetal bovine serum (FBS) and Lipo2000 were from Thermo fisher scientific (USA). Penicillin and streptomycin are from Solarbio life sciences (China).

### SXNI constituent analysis

SXNI was analysed through liquid chromatography which was performed on a Shimadzu inertsil ODS-3 C18 column (5 µm, 250 mm × 4.6 mm), at a flow rate of 1 mL/min using high-performance liquid chromatography (HPLC) on a Waters 2695 system (Waters, USA), and the detection wavelength was 360 nm. The mobile phase A was acetonitrile, and B was water containing 0.4% phosphoric acid. The gradient profile for the LC pumps under the final chromatography conditions are illustrated in Table 1.

**Table 1.** The solvent gradient profile of the HPLC pumps.

Min	A (%)	B (%)
0	15	85
8	15	85
17	17	83
25	17	83
34	20	80
40	20	80
70	35	65
70.01	15	85

### Evaluation of vascular leakage

The dose of SXNI recommended in clinical setting is 1.4 mg/kg, which convert to the animal (mouse) equivalent dose is 17.22 mg/kg according to FDA guidance (Nair and Jacob 2016). In the present study, mice were given with 14 mg/kg (SXNI/1, equal with 0.8 times of human clinical dose) or 70 mg/kg (SXNI/4, equal with 4 times of human clinical dose) SXNI. Naive mice were treated (i.v.) with negative control combined with EB (CTL-EB) or SXNI combined with EB (SXNI-EB). Vascular leakage was analysed by evaluating the ear area of blue colour 30 min after drug treatment. The score criterion is the same as previous reports (Han et al. 2016). The EB content was extracted from ears preserved in formamide.

### Histopathology examination

Mouse ears and lungs were fixed in 4% paraformaldehyde, embedded in paraffin, sectioned at a thickness of 3 μm, and then stained with haematoxylin and eosin (H&E) for morphological evaluation.

### Cell culture

Human umbilical vein endothelial cells (HUVECs) were obtained from American Type Culture Collection (ATCC, Manassas, VA, United States). The cells were cultured in DMEM supplemented with 10% foetal bovine serum, penicillin (100 U/mL) and streptomycin (50 μg/mL) in an incubator with a humidified atmosphere of 5% CO_2_ and 95% air at 37 °C.

### RNA interference

The siRNA oligonucleotides were purchased from GenePharma (Shanghai, China). Cells seeded in 6-well plates were transfected with siRNAs (200 nM) in Lipo 2000. Cell lysate was collected for immunoblotting analysis 48 h later. The siRNA target sequences used are as follows: negative control, 5′-UUCUCCGAACGUGUCACGUTT-3′, TSC1 (Human), 5′-CCAAAUCUCAGCCCGCUUUTT-3′.

### Assessment of endothelial permeability using transwell assay

Endothelial permeability was assessed through FITC-dextran flux measurements which were reported previously (Han et al. 2016, 2018). Briefly, HUVECs were cultured in a transwell chamber until the formation of monolayer. The cells were treated with SXNI (0.14, 0.28, or 0.7 mg/mL) for 1 h, or pre-treated with 10 nM rapamycin for 18 h and then stimulated with 0.7 mg/mL SXNI for 1 h. The fluorescence of FITC-dextran was detected through Thermo Scientific Varioskan Flash microplate reader.

### Fluorescence staining

HUVECs were treated with SXNI (0.14, 0.28, or 0.7 mg/mL) for 1 h, or pre-treated with 10 nM rapamycin for 18 h and then stimulated with 0.7 mg/mL SXNI for 1 h. Cells were stained with 5 mg/mL rhodamine-phalloidin for 1 h and visualized using Olympus IX71 fluorescent microscope.

### Immunoblot analysis

Whole cells were lysed in lysis buffer [2% SDS, 10% glycerol, 10 mM Tris (pH 6.8), and 100 mM DTT], tissues were homogenized in water using FastPrep-24 (MP biomedicals, USA) for 20 sec, and then diluted with 4 × lysis buffer. Samples were boiled at 98 °C for 10 min, and then subjected to immunoblotting as previously described (Ma et al. 2014; Wang et al. 2016).

### Statistical analysis

These data are shown as the mean ± SEM. Comparisons between groups were performed using 2-tailed Student’s *t*-tests. Statistical analyses were performed using Prism 8.0 software (Graph-Pad software Inc.), and *p* values less than 0.05 were considered significant.

## Results

### SXNI induced pseudo-allergic reactions in mice

**Figure 1.**
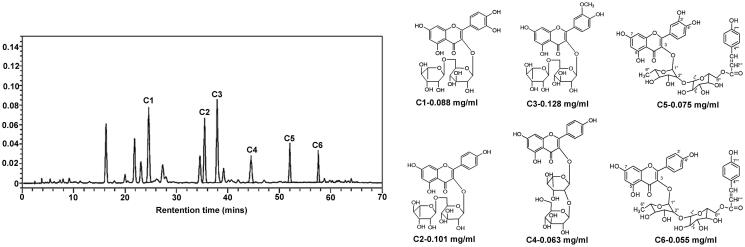
HPLC analysis of Shuxuening injection.

SXNI was analysed through HPLC system. The components including rutin (C_27_H_30_O_16_), kaempferol-3-*O*-rutinoside (C_27_H_30_O_16_), isorhamnetin-3-*O*-rutinoside (C_28_H_32_O_16_), kaempeerol-3-2″-*O*-glucorhamnoside (C_27_H_30_O_15_), quercetin 3-*O*-2″-(6‴-*p*-coumaroyl) glucosyl rhamnoside (C_36_H_36_O_18_), kaempferol 3-*O*-2″-(6‴-*p*-coumaroyl) glucosyl rhamnoside (C_36_H_36_O_17_) were identified in SXNI, and the contents are 0.088, 0.101, 0.128, 0.063, 0.075 and 0.055 mg/mL respectively (Figure 1).

**Figure 2.**
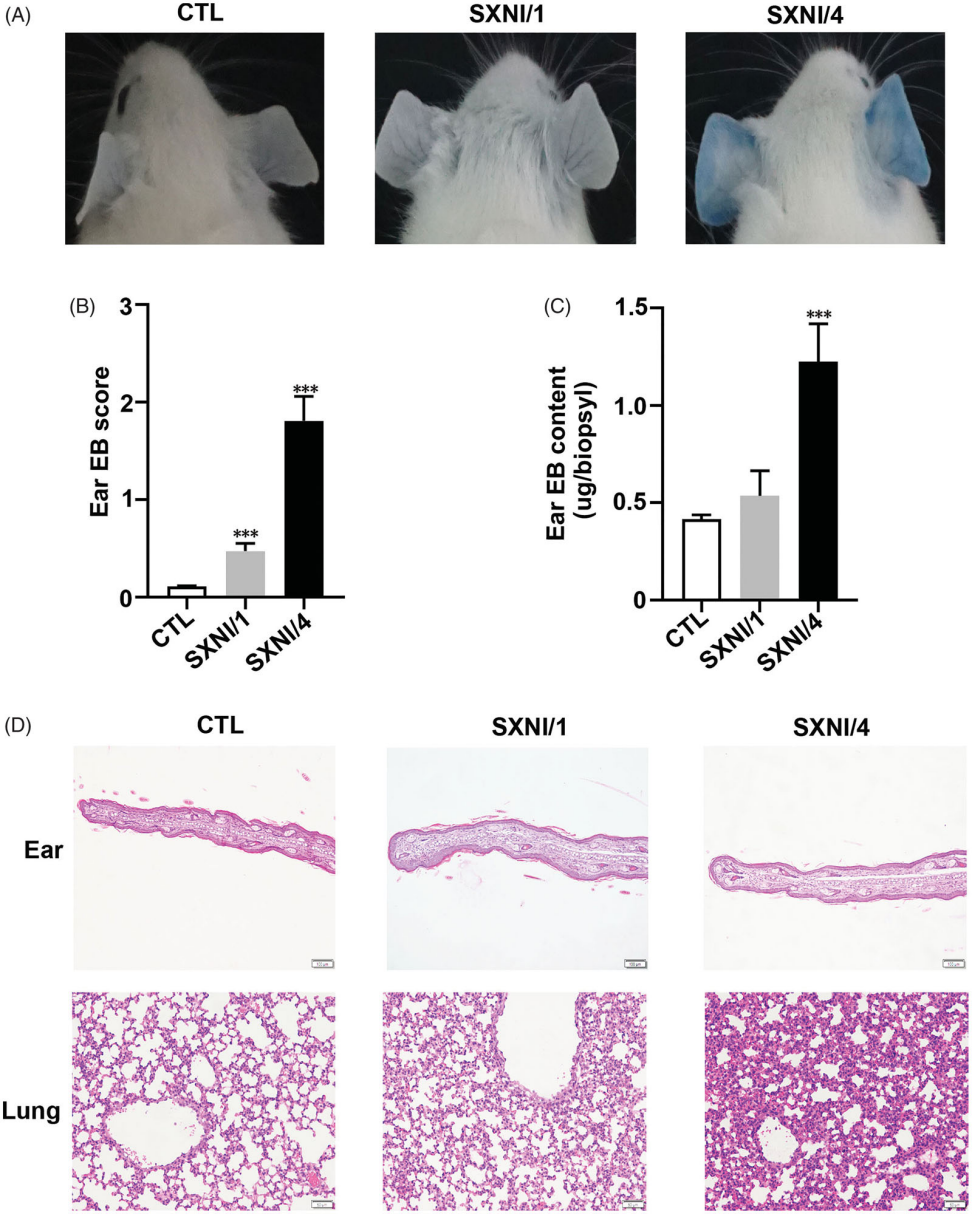
SXNI induces pseudo-allergic reactions through elevation of vascular permeability in mice. (A) Representative mouse ears. (B) Score of EB extravasation in the mice ear. (C) The concentration of EB extravasation in the mice ear. *n* = 10 in A–C. (D) Representative H&E-stained mouse ear and lung sections. *n* = 5. Scale bar = 100 μm in ear, Scale bar = 50 μm in lung. CTL: control; EB: Evans blue; SXNI: Shuxuening Injection; SXNI/1, 14 mg/kg SXNI; SXNI/4, 70mg/kg SXNI.

EB binds to plasma albumin to form albumin-EB complex which is widely used as a marker to evaluate vascular leakage. As we known, alterations of vascular permeability were one of these reasons to mediate pseudo-allergic reactions (Han et al. 2016, 2018). The recommended clinical dose of SXNI is 1.4 mg/kg, which convert to the animal (mouse) equivalent dose is 17.22 mg/kg according to FDA guidance (Nair and Jacob 2016). In the present study, mice were given with 14 mg/kg (SXNI/1, equal with 0.8 times of human clinical dose) or 70 mg/kg (SXNI/4, equal with 4 times of human clinical dose) SXNI. Naive mice were treated (i.v.) with negative control combined with EB (CTL-EB) or SXNI combined with EB (SXNI-EB). Ear EB extravasation initiated at 10 min after drug administration, and turned to max value at 30 min since drug treatment. All cohorts of mice were collected to evaluated vascular leakage 30 min after drug treatment. SXNI/4 enhanced vascular permeability demonstrated by the augmentation of ear EB concentration compared to CTL mice (Figure 2(A–C)). Slight edoema and microvascular dilation in the ears, and broaden of alveolar septal and monocyte infiltration in the lungs were observed in SXNI/4 group of mice (Figure 2(D)). On the other hand, SXNI/1 did not mediate visible histopathological alterations in the ears and lungs.

### mTOR mediates vascular endothelial cell hyperpermeability

**Figure 3.**
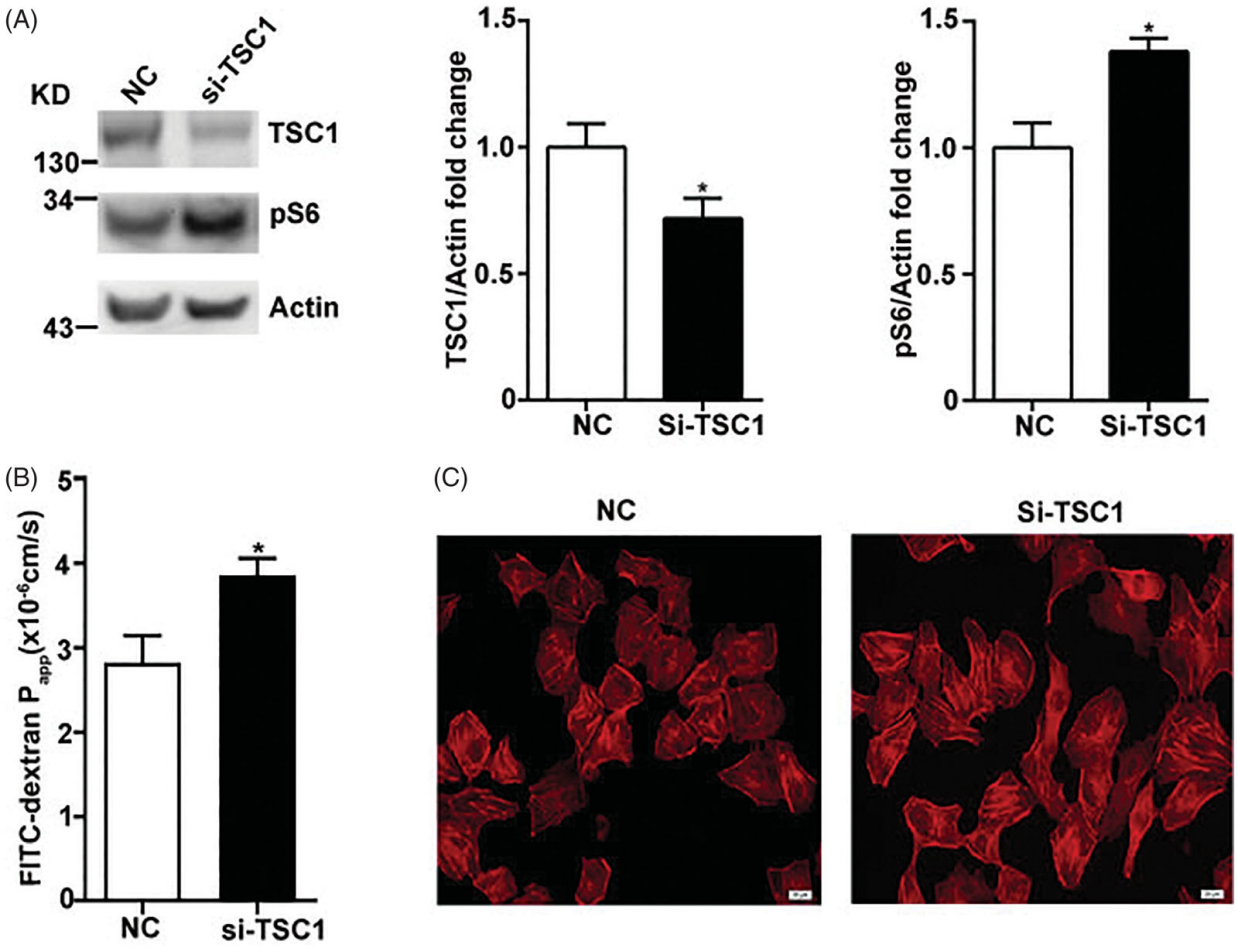
mTOR hyperactivation enhances vascular endothelial cell hyperpermeability in HUVECs. (A) Immunobloting and quantification of protein expression with the antibodies as indicated in HUVECs transfected with si-TSC1 or negative control (NC). (B) Fluorescence apparent permeability coefficient of FITC-dextran through the HUVEC monolayer (C) F-actin morphology in HUVEC monolayer. Scale bar = 20 μm. *n* = 4.

mTOR plays pivotal role in maintaining the status of vascular endothelial cell (Ma et al. 2014). Transwell assay is widely used in analysing vascular permeability by evaluating the diffusion of FITC-dextran through a confluent endothelial monolayer. TSC1 is a suppressor of mTOR, knockdown of *TSC1* actives mTOR signalling pathway as indicated by the augmentation of pS6 (Figure 3(A)). Transwell assay was performed in HUVEC-si-TSC1 cells and the negative control cells (NC). The permeability coefficient of FITC-dextran significantly increased in si-TSC1 cells in contrast to NC cells (Figure 3(B)). Cells were stained with rhodaminephalloidin to visualize F-actin. Few thin F-actin stress fibres sporadically displayed around in the NC cells, conversely, mTOR hyperactivation turned F-actin to thicker stress fibres diffused throughout the cells (Figure 3(C)). Therefore, we conclude that mTOR hyperactivation increases vascular endothelial cell hyperpermeability.

### SXNI stimulates vascular endothelial cell hyperpermeability and hyperactives mTOR

**Figure 4.**
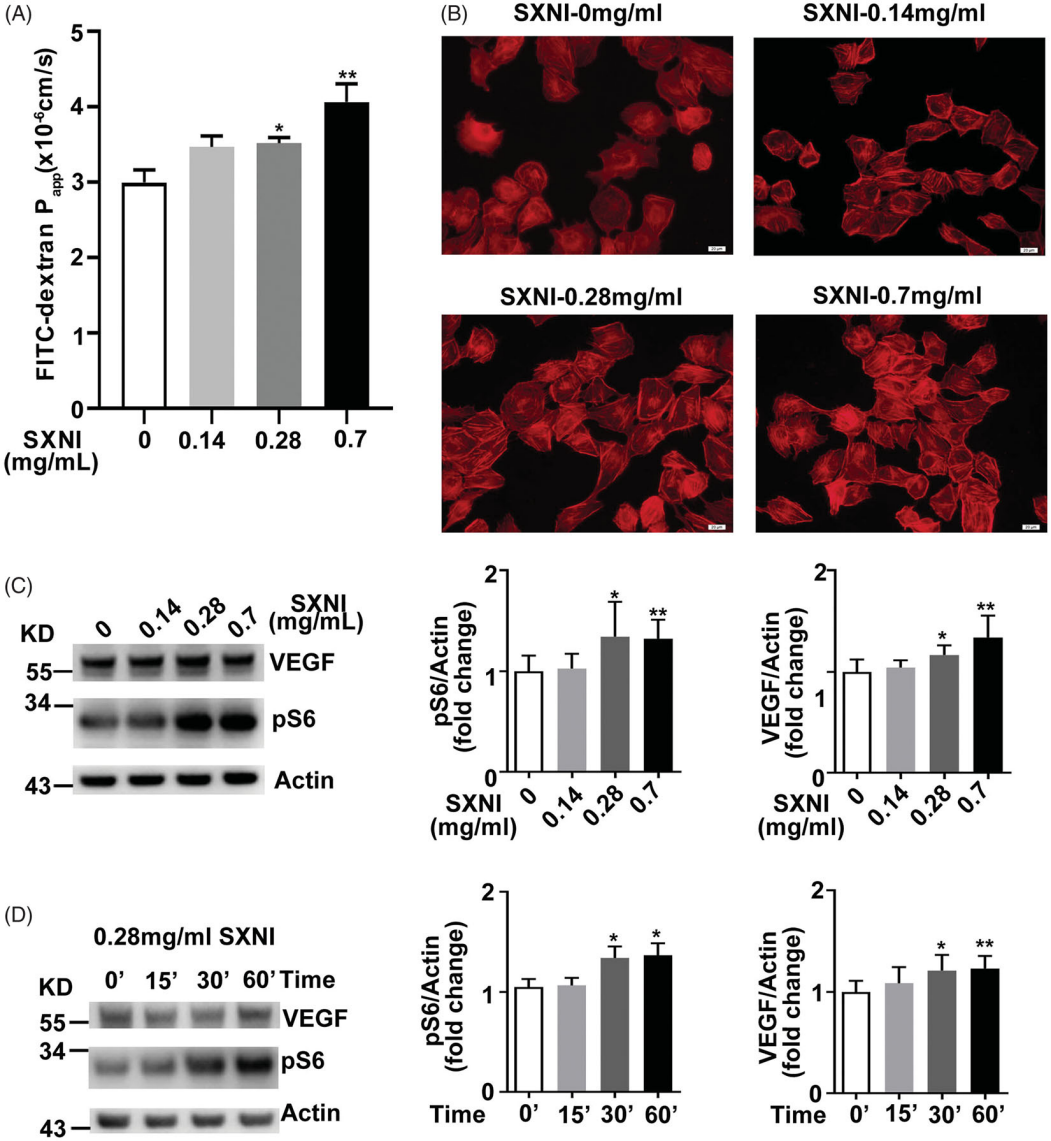
SXNI stimulates vascular endothelial cell hyperpermeability and actives mTOR signaling pathway in HUVECs. (A) Fluorescence apparent permeability coefficient of FITC-dextran through the HUVEC monolayer. (B) F-actin morphology in HUVEC monolayer. Scale bar = 20 μm. (C–D) Immunobloting and quantification of protein expression with the antibodies as indicated. *n* = 4. SXNI: Shuxuening Injection.

We demonstrated SXNI induced pseudo-allergic reactions in mice. However, the mechanism is unknown. We treated HUVECs with 0.14, 0.28, and 0.7 mg/mL SXNI for 1 h, and then analysed the alterations of cell monolayer permeability and F-actin morphology. SXNI is used at a concentration of 0.14 mg/mL to treat patients in clinics. The permeability coefficient of FITC-dextran increased dramatically in the condition of SXNI treatment at these concentrations of 0.28 and 0.7 mg/mL (Figure 4(A)). In addition, F-actin morphology formed thicker stress fibres after SXNI administration at these concentrations of 0.28 and 0.7 mg/mL (Figure 4(B)). Thus, SXNI stimulates vascular endothelial hyperpermeability *in vitro*.

Augmentation of pS6 is a marker of hyperactivation of mTOR signalling pathway. We treated HUVECs with 0.14, 0.28, and 0.7 mg/mL SXNI for 30 min and found the expression level of pS6 and VEGF increased at these groups of SXNI 0.28 and 0.7 mg/mL compared to the SXNI 0 mg/mL group (Figure 4(C)). Furthermore, we treated HUVECs with 0.28 mg/mL SXNI for 15, 30, and 60 min, respectively, and observed that the expression of pS6 and VEGF increased time dependently (Figure 4(D)). Collectively, SXNI induces mTOR hyperactivation in a dose and time dependent way.

**Figure 5.**
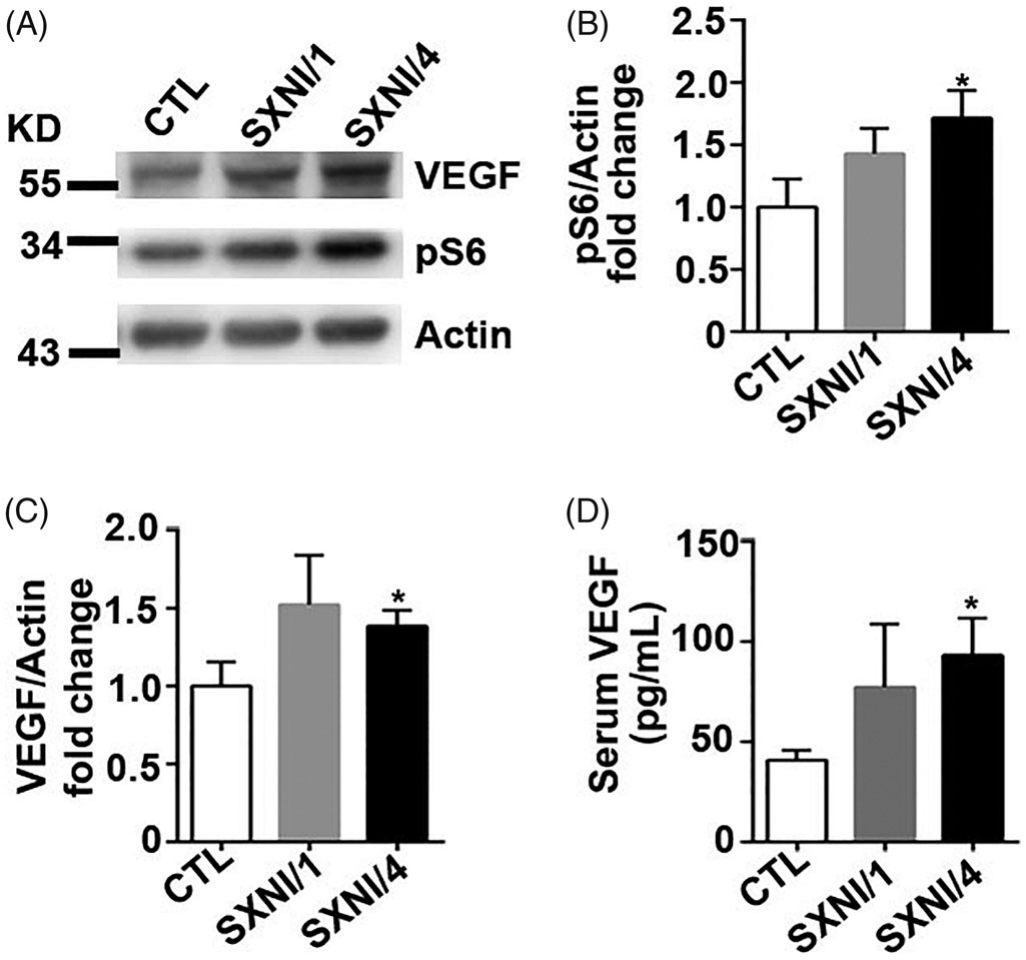
SXNI activates mTOR signaling pathway in mice. (A–C) Immunobloting and quantification of protein expression of mouse ears with the antibodies as indicated. *n* = 4 in A–C. (D) Serum VEGF level in each cohort of mice. *n* = 5. SXNI: Shuxuening Injection.

Additionally, we detected the status of mTOR signalling pathway in mice after SXNI administration. The expression level of pS6 and VEGF increased in the SXNI/4 cohort compared to the CTL mice (Figure 5(A–C)). Moreover, serum VEGF raised in SXNI/4 group in contrast to CTL group (Figure 5(D)). In a word, SXNI induces mTOR signalling pathway activation both *in vitro* and *in vivo*.

### Rapamycin alleviates SXNI mediated vascular endothelial cell hyperpermeability both in vitro and in vivo

**Figure 6.**
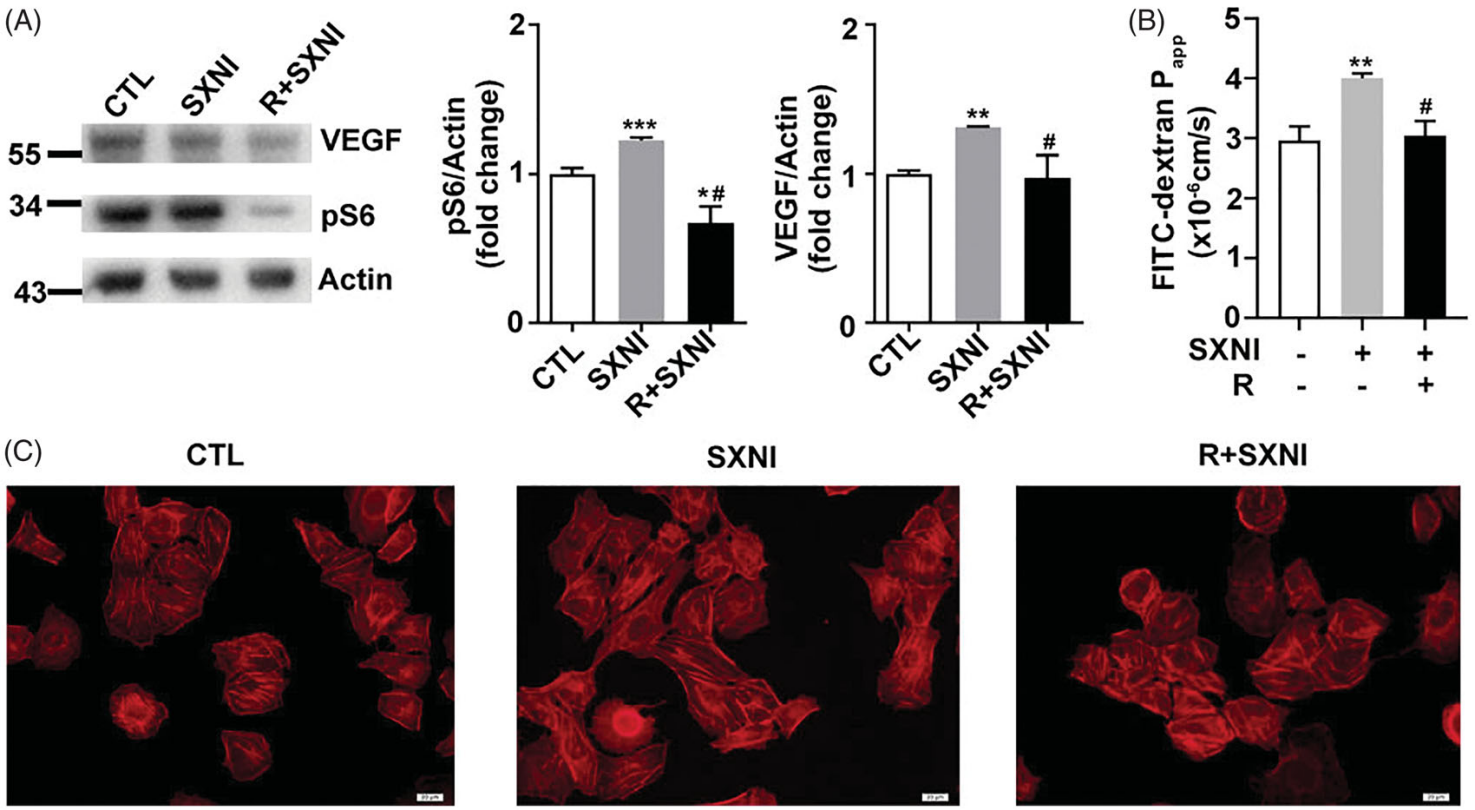
Rapamycin alleviates SXNI mediated vascular endothelial cell hyperpermeability. (A) Immunobloting and quantification of protein expression with the antibodies as indicated. (B) Fluorescence apparent permeability coefficient of FITC-dextran through the HUVEC monolayer. (C) F-actin formation and distribution in HUVEC monolayer. Scale bar = 20 μm. *n* = 4. CTL: control; R: rapamycin; SXNI: Shuxuening Injection.

Rapamycin is a specific inhibitor of mTOR. We pre-treated HUVECs with 10 nM rapamycin for 18 h and treated cells with SXNI (0.7 mg/mL) for 1 h, then observed the alterations of mTOR signalling pathway, cell monolayer permeability and F-actin morphology. Immunoblotting of cell lysis showed rapamycin decreased the expression of VEGF and pS6 induced by SXNI (Figure 6(A)). Rapamycin deduced permeability coefficient of FITC-dextran stimulated by SXNI (Figure 6(B)), and modified thicker stress fibres of F-actin caused by SXNI (Figure 6(C)).

**Figure 7.**
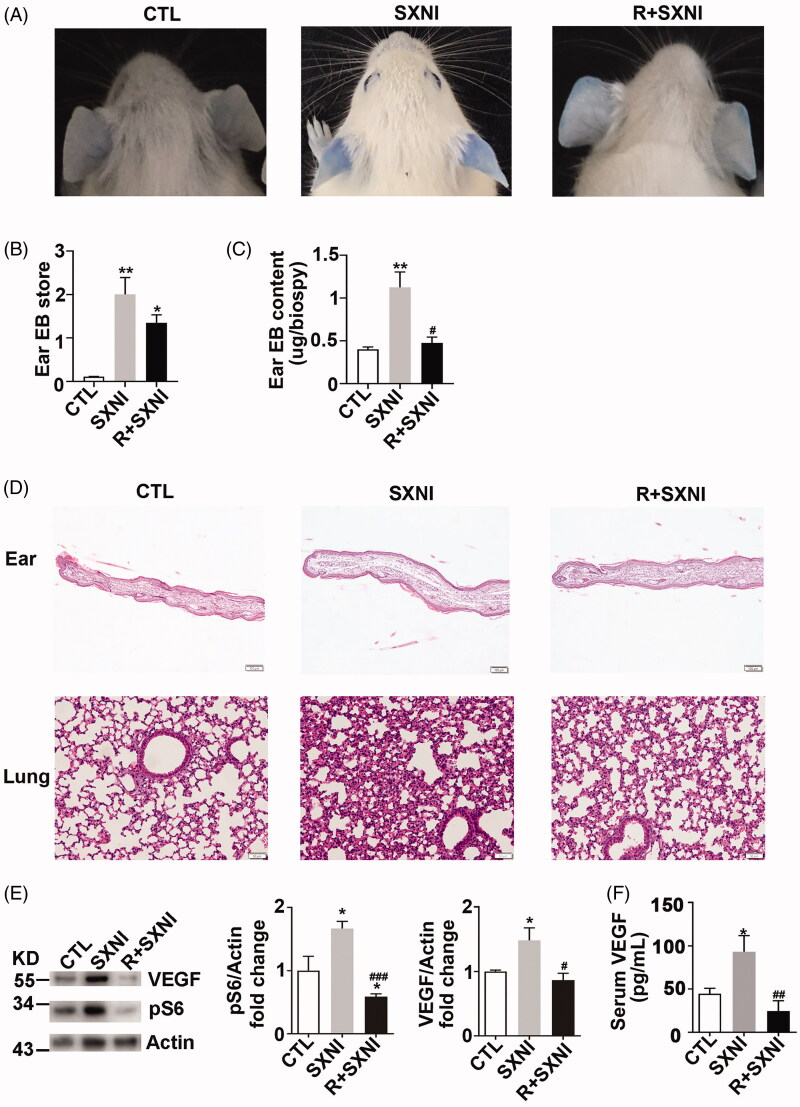
Rapamycin alleviates SXNI mediated vascular permeability in mice. (A) Representative mouse ears. (B) Score of EB extravasation in the mice ear. (C) The concentration of EB extravasation in the mice ear. *n* = 10 in A–C. (D) Representative H&E-stained mouse ear and lung sections. *n* = 5. Scale bar = 100 μm in ear, Scale bar = 50 μm in lung. (E) Immunobloting and quantification of protein expression of mouse ears with the antibodies as indicated. *n* = 4. (F) Serum VEGF level in each cohort of mice. *n* = 5. CTL: control; EB: Evans blue; SXNI: Shuxuening Injection.

Naive mice were pre-treated with rapamycin (2 mg/kg, ip) for 2 days and subjected to SXNI (70 mg/kg)/EB. All groups of mice ears were collected to evaluated vascular leakage. SXNI increased EB extravasation in contrast to CTL mice, rapamycin dramatically decreased SXNI induced EB extravasation (Figure 7(A–C)). Pathological analysis revealed that rapamycin reduced SXNI mediated ear edoema and microvascular dilation, and decreased SXNI induced alveolar septal and monocyte infiltration in the lung (Figure 7(D)). In addition, rapamycin decreased ear protein expression of VEGF and pS6, and the level of serum VEGF induced by SXNI (Figure 7(E–F)). Taken together, these results show that rapamycin alleviates SXNI mediated endothelial hyperpermeability both *in vitro* and *in vivo*.

## Discussion

We demonstrated that SXNI induced pseudo-allergic reactions in this study. As we known, alteration of vascular permeability is one of these reasons to mediated pseudo-allergic and allergic reactions (Han et al. 2016, 2018). In the current manuscript, we found SXNI mediated vascular permeability through hyper-activating mTOR. Collectively, our results demonstrate that SXNI stimulates pseudo-allergic reactions through hyperactivating mTOR.

SXNI is a prescribed drug in Chinese hospitals and clinics to treat cardiocerebral vascular system related disease (Wang et al. 2019; Cao et al. 2020). Nevertheless, the use of SXNI is related to the risk of ADRs in clinics, and 68.4% of SXNI mediated ADRs are hypersensitivity reactions (Mao et al. 2016). The ADRs of SXNI were specifically listed in the *China State Food and Drug Administration Annual Adverse Reactions Report for Drugs* during 2013–2016. The researches of SXNI focus on the clinical application and quality control. Drug hypersensitivity reactions (DHR) are classified as IgE-mediated drug allergies, T cells-induced drug allergies and pseudo-allergic reactions. Pseudo-allergic reactions are non-immune-mediated hypersensitivity reactions. Our lab reported that SXNI induced non-IgE hypersensitivity reactions for the first time (Yi et al. 2017). Pseudo-allergic reactions stimulate indistinguishable clinical manifestation with immune-mediated hypersensitivity reactions. Drugs including acetylsalicylic acid, diclofenac, mefenamic acid, ibuprofen, metamizole, ciprofloxacin, moxifloxacin, norfloxacin, iomeprol, iodihexol, rocuronium, suxamethylcholin, vancomycin, penicillin and shuanghuanglian injection initiate pseudo-allergic reactions (Han et al. 2016, 2018; Pichler 2019). Hyeractivation of RhoA/ROCK signalling pathway involves in drug induced pseudo-allergic reactions (Han et al. 2016, 2018, 2019). Mast cell specific receptors comprising MrgprB2 and MrgprX2 are pivotal for pseudo-allergic drug reactions (McNeil et al. 2015). In this study, we identified mTOR involved in SXNI stimulated pseudo-allergic reactions.mTOR a central orchestrator of cellular metabolism, involves in innate and adaptive immune response (Zou et al. 2019). mTOR regulates bacterial stimulated inflammatory responses in monocytes, macrophages, and primary dendritic cells (Weichhart et al. 2008). mTOR modulates T cell proliferation and differentiation (Delgoffe et al. 2009, 2011; Zou et al. 2019). mTOR is engaged in food allergens by regulating Th2-regulated immune responses (Fu et al. 2017). mTOR controls lung allergic inflammation through modulating metabolic adaptation of antigen-presenting cells subsets (Sinclair et al. 2017). mTOR specific inhibitor rapamycin, a bacterial macrolide, is used in transplantation, and to treat tuberous sclerosis and lymphangioleiomyomatosis clinically (Xu et al. 2018; Lechuga and Franz 2019; Park et al. 2019; Kim et al. 2020). Rapamycin was reported to prevent house dust mite (HDM)-mediated allergic asthma and ovalbumin-induced asthma (Eynott et al. 2003; Mushaben et al. 2011). Inhibition of PI3K/Akt/mTOR/HIF-1α/VEGF signalling pathway is benefit for attenuating allergic airway inflammation (Choi et al. 2013). However, the function of mTOR-VEGF pathway in drug mediated pseudo-allergic reactions is unknown. Our work indicates that mTOR-VEGF pathway is hyperactivated in SXNI induced pseudo-allergic reactions, and rapamycin might be a potentially drug to prevent SXNI mediated pseudo-allergic reactions.

## Conclusions

SXNI stimulated pseudo-allergic reactions through hyeractivation of mTOR signalling pathway. Rapamycin might be a potentially drug to prevent and treat SXNI mediated pseudo-allergic reactions.
